# A Systematic Review of Polyvictimization among Children with Attention Deficit Hyperactivity or Autism Spectrum Disorder

**DOI:** 10.3390/ijerph16132280

**Published:** 2019-06-27

**Authors:** Lisa Hellström

**Affiliations:** Department of School Development and Leadership, Malmö University, 211 19 Malmö, Sweden; lisa.hellstrom@mau.se

**Keywords:** attention deficit hyperactivity disorder, autism spectrum disorder, children, polyvictimization

## Abstract

Children with Autism Spectrum Disorder (ASD) or Attention Deficit Hyperactivity Disorder (ADHD) have shown an increased risk for violence and victimization. However, research on exposure to multiple forms of victimization in different contexts are scarce. Hence, the current aim is to review the evidence about polyvictimization among children with ASD or ADHD. PsycInfo, ERIC, ERC, Scopus, and PubMed databases were systematically searched until 12 March 2019 to identify empirical studies with reported prevalence rates of at least four forms of victimization among children with ASD or ADHD. A total of 6/1300 articles were included in the review, ranging in sample sizes from 92 to 4114. The reported prevalence rates for polyvictimization were 1.8% and 23.1% for children with ASD and 7.3% for children with ADHD. The results emphasize the high prevalence of violence and victimization, including polyvictimization, among children with ASD or ADHD. Polyvictimization among children with ASD or ADHD is a highly under researched area. Significant knowledge gaps and important methodological considerations that provide important implications for future research include lack of information on cyber bullying, frequency or intensity of victimization, and the failure to include children as informants and to report health outcomes associated with polyvictimization.

## 1. Rationale

In many aspects related to victimization and health, children with Autism Spectrum Disorder (ASD) and Attention-Deficit/Hyperactivity Disorder (ADHD) is a vulnerable group. This includes aspects in the contexts of school, home, and community and takes place face-to-face as well as on the internet. ASD and ADHD are the two most common forms of neurodevelopmental disorders in the world, affecting around 5–7% (ADHD) and 1–4% (ASD) of children [1,2]. According to the American Psychiatric Association [3], ADHD symptoms are divided into two categories of inattention and hyperactivity and impulsivity that include behaviors such as failure to pay close attention to details, difficulty organizing tasks and activities, excessive talking, fidgeting, or an inability to remain seated in appropriate situations. These symptoms may be present in multiple settings (e.g., school and home). Further, people with ASD tend to have communication deficits, such as responding inappropriately in conversations, misreading nonverbal interactions, or having difficulty building friendships appropriate to their age. ASD, as used in the present study, includes autistic disorders including Asperger’s syndrome according to the “Diagnostic and Statistical Manual of Mental” Disorders [3]. Overlapping features of ASD and ADHD include attention deficit and overactivity, behavior problems and difficulty with social skills [4]. Both disorders can have long-term negative impacts on academic achievement, work performance, and mental health [5,6]. Due to their stressful life situation, children with ASD or ADHD may be more susceptible to multiple forms of victimization across different contexts such as school, home, and their community.

### 1.1. Vulnerability to Different Forms of Victimization

Compared to typically developing (TD) children, children with ASD and ADHD are more likely to be victims of peer victimization, child maltreatment, and other forms of victimization. Polyvictimization is used as a concept to examine vulnerability to multiple forms of victimization experienced in different contexts. However, little is known about polyvictimization among children with specific forms of disability, such as ASD or ADHD [7]. Polyvictimization is defined as exposure to multiple forms of abuse, victimization, or violence including, but not limited to, conventional crime, child maltreatment, peer and sibling victimization, sexual assault, and witnessing and indirect victimization [8]. Polyvictimization can also be expressed as exposure to violence in different settings or environments, such as the school, home, and community. Examining polyvictimization could help account for large variations in the traumatic symptoms seen in children subjected to various forms of childhood adversity [9]. Due to their stressful life situation, it is of particular importance to understand the vulnerability of children with ASD or ADHD and the intersections of violence and victimization across these different contexts and domains of exposure [10].

Peer victimization is defined as repetitive and intentional aggressive behavior that is systematic and involves inequality in power between the perpetrator(s) and the victim [11]. Peer victimization can take place ‘face-to-face’ or via electronic media [12]. Approximately 44% of school-aged children with ASD report peer victimization, where verbal bullying is the most common form of bullying reported [13]. In the USA, prevalence rates as high as 94% have been reported by mothers of children with ASD [14]. Children and adolescents with Asperger’s syndrome and/or with milder deficits in social understanding, early adolescents, those attending mainstream school, and those with concurrent behavioral difficulties are those most at risk of peer victimization. The inclusion of children with ASD in mainstream schools are becoming increasingly more common and there is concern that this affects their involvement in peer victimization and their school performance negatively due to the symptoms associated with the disabilities [5,15]. While a mainstream school setting offers important opportunities for growth in social competency for adolescents with ASD, it may also increase the probability of negative peer interactions and experiences [16]. However, the results regarding the impact of school setting and the risk for peer victimization among students with ASD are conflicting [17,18].

Adolescents with ADHD is a group with an increased risk of peer victimization compared to TD youth. Research on ADHD has mostly been based on male samples, with focus on girls emerging relatively recently. Compared to TD youth, girls with ADHD demonstrate higher levels of peer rejection, which in turn predict poorer social adjustment and a host of problem behaviors [19]. For young adolescents with ADHD, Becker et al., [6] found that 51% of students ages 11–15 reported relational victimization but found no sex differences regarding relational or reputational victimization. However, males with ADHD reported more physical victimization than females. The risk of exposure to peer victimization as well as abuse and maltreatment in the home are elevated among children with chronic health conditions. This means that frequent peer victimization warrants particular vigilance as it appears to be an indicator of severe violence at home [20].

Child maltreatment, which is also referred to as child abuse or neglect, is defined as “all forms of physical and/or emotional ill-treatment, sexual abuse, neglect, negligence, and commercial or other exploitation, which results in actual or potential harm to child’s health, survival, development or dignity in the context of a relationship of responsibility trust or power. Exposure to intimate partner violence is also sometimes included as a form of child maltreatment” [21]. Children with disabilities are more likely to be victims of child abuse than their TD peers, where higher levels of disability are associated with an increased risk of abuse [22]. Chronically maltreated children also appear to be at high-risk for developing clinical levels of problems [23]. Further, children with ASD have shown to be between two to three times more likely to experience maltreatment compared to population controls, with a substantial elevated risk for physical abuse [24]. A follow-up study of maltreatment in girls with ADHD found that twice as many girls with ADHD (22.9%) reported at least one form of maltreatment at a five-year follow-up compared to girls without ADHD (11.4%) [25].

Further, ADHD symptoms of the inattentive types have shown associations with supervision neglect, physical neglect, and physical and contact sexual abuse, while the hyperactive types of symptoms have shown an increased risk for supervision neglect and physical abuse [26]. Parents of children with ADHD may experience increased levels of parenting stress compared to parents of TD children, with predictors including child externalizing and internalizing problems, maternal depressive symptoms, parental ADHD diagnosis, and low marital quality [27]. It has been suggested that children with disabilities may be at greater risk for maltreatment because their extensive needs and/or behavioral difficulties can trigger maladaptive responses in some parents [28].

Other forms of victimization commonly reported among children and adolescents include violence in the community and in the neighborhood, both own exposure to violence and witnessing different forms of violence. Exposure to violence in combination with witnessing violence have shown to increase the risk of internalizing and externalizing problems, as well as child and adult health risk indicators [29]. There is robust evidence that exposure to interparental aggression is associated with significant disruptions in children’s psychosocial functioning, especially among preschoolers [30]. Being both victim-of and witness-to violence is significantly associated with ADHD symptoms, particularly among girls [31]. Further, a study by Brown et al. [32] found that children with ADHD reported a significantly higher prevalence of nine different types of adverse childhood experiences (ACE) compared to children without ADHD, including neighborhood violence (20.1% vs. 9.5%; *p* < 0.001) and domestic violence (15.2% vs. 6.4%; *p* < 0.001). Similar results were found by Fuller-Thomson and Lewis [33] who used a retrospective design to explore the relationship between early adversities and ADHD. Their results showed that for both men and women, childhood sexual abuse was significantly related to higher odds of ADHD (men OR = 2.57, *p* < 0.001; women OR = 2.55, *p* < 0.001), while domestic violence was only associated with elevated odds of ADHD among women (OR = 1.54, *p* < 0.03).

### 1.2. Polyvictimization

In 2005, Finkelhor, Ormrod, Turner, and Hamby [8] introduced the construct of “polyvictimization” arguing that more comprehensive investigations of violence against children were required, so that the experiences of those affected by multiple forms of violence were understood accurately [34]. There are reasons to believe that children who are exposed to one form of victimization also experience other forms of victimization [35,36,37]. Focusing on only one or a few forms of child victimization is likely to substantially underestimate the full burden of victimization exposure and the full strength of the relationship between victimization and child mental health [38]. Further, even low-frequency polyvictimization appears to have a more severe impact than high frequency single-victimization [9], which supports polyvictimization as a unique and important construct for schools and other authorities to understand the whole situation of the victims. Despite research showing an increased risk for victimization among children and adolescents with ASD and/or ADHD, very few studies have studied the vulnerability of children with ASD and/or ADHD of exposure to multiple forms of victimization. Ford, Wasser, and Connor [39] studied polyvictimization among psychiatrically impaired children and found that 22.8% among 259 outpatient admissions had experienced at least two forms of victimization. Of these, 83% reported sexual abuse, 78% reported physical abuse, and 86% reported other trauma history.

As polyvictimization has been associated with mental ill-health and psychiatric symptoms [10,40,41,42], it is important to study this phenomenon further, especially among the vulnerable group of children and students with ASD and ADHD. Due to their stressful life situation, children with ASD or ADHD may be more susceptible to multiple forms of victimization. Hence, the focus in this systematic review is to highlight the vulnerability of victimization in different contexts, i.e., in school, at home, and in the community. This review does not seek to separate cyber victimization as a different arena for victimization as it could be a tool for victimization in school, at home, as well as in the community.

### 1.3. Study Design and the Use of Different Informants

Research results on prevalence rates of victimization among children and youth with ASD and/or ADHD are ambiguous. One explanation of the great variation in prevalence rates could be the various forms of gathering this data where different instruments and informants are used. The main research tool used to measure polyvictimization to date has been the Juvenile Victimization Questionnaire (JVQ). The JVQ includes 34 forms of offenses against youth that cover five general areas of concern (1) Conventional crime (eight questions, e.g., robbery), (2) Child Maltreatment (four questions including physical, emotional and neglect), (3) Peer and Sibling Victimization (six questions, e.g., bullying), (4) Sexual victimization (seven questions including peer or adult perpetration), and (5) Witnessing and Indirect Victimization (nine questions, e.g., witnessing family violence, witnessing an assault with a weapon) [43]. When presenting the results from studies using JVQ, the results will be presented according to these five areas. While self-reports are the most commonly used method for gathering data on peer victimization among TD, parental reports [14,44] or teacher reports [45] are the most common among children with ASD. It has been suggested that this may be based on the belief that children and youth with ASD are unable to answer questions on peer victimization due to their inability to understand complex social situations [46]. Experiences with peer victimization as reported by ASD youth themselves may differ from those typically measured in questionnaires, indicating that their personal experiences and perceptions of such victimization are not well understood [47]. Research involving both parental, and self-reports have found no differences in reported prevalence rates regarding cyberbullying [48], while other research has used different questions for parents and their children [49]. A comparison of teacher and parent reports shows that parents tend to rate their child as a victim of bullying to a higher degree than teachers, which is likely to reflect the different environments in which teachers and parents see children [50]. Adams, Fredstrom, Duncan, Holleb, and Bishop [51] tested the association between peer victimization and internalizing symptoms in adolescent males with ASD using self-reports. Their results showed that the self-report measure was valid and reliable and associated with internalizing symptoms even after controlling for parent reports of peer victimization. This highlights the importance of also using children themselves as informants instead of solely relying on parental reports.

## 2. Objectives

To be able to enhance the knowledge and improve the life situation for children with ASD and ADHD, the aim with the current article is to review the evidence about polyvictimization among children with Attention Deficit Hyperactivity Disorder (ADHD) or Autism Spectrum Disorder (ASD). Research questions are: (i) to what degree are children with ASD or ADHD exposed to polyvictimization, (ii) what form of victimization is most commonly reported, and (iii) do the prevalence rates of victimization vary between different study characteristics such as type of informant and study design.

## 3. Methods

### 3.1. Protocol and Registration

This review was registered (registration number: CRD42019128779) with the PROSPERO international prospective register of systematic reviews, the Centre for Reviews and Dissemination, University of York, and the review protocol published on this database on 15 April 2019.

### 3.2. Eligibility Criteria

In this review, polyvictimization is defined as exposure to multiple forms of abuse, victimization, or violence, including, but not limited to, conventional crime, child maltreatment, peer and sibling victimization, sexual assault, and witnessing and indirect victimization [8]. This review does not treat cyber victimization as a separate form of victimization, merely as a tool for different forms of victimization [52]. The word polyvictimization (by definition, exposure to multiple forms of victimization) was chosen in this review and the included studies must investigate at least four different forms of victimization. Information about (i) the number of different forms of victimization, (ii) the frequency of victimization, (iii) background information for example type of disability/disabilities, age, sex, as well as (iv) study design and assessment methods used, are sought for the included articles.

#### 3.2.1. Inclusion Criteria

Studies that have investigated prevalence rates of at least four different forms of victimization including children or adolescents with ASD or ADHD. A cutoff point of four forms of victimization to define polyvictimization was chosen for consistency with previous studies [36,53,54]. Children and adolescents are defined as people up to 19 years of age. The samples could be drawn from clinical or population-based settings. Types of victimization included are conventional crime, child maltreatment, peer and sibling victimization, sexual assault, and witnessing and indirect victimization.

#### 3.2.2. Exclusion Criteria

This review aimed to establish prevalence rates of polyvictimization among children and adolescents with ASD or ADHD. Studies reporting in languages other than English or Swedish, investigating children with disabilities other than ASD or ADHD, investigating fewer than four forms of victimization, or studies with a target sample older than 19 years of age are excluded in the review.

### 3.3. Information Sources and Search Strategy

Relevant studies were identified using two search strategies. First, an online search was performed in five databases: PsycInfo, ERIC, ERC, Scopus, and PubMed, until 12 March 2019. Search terms consisted of three main concepts related to the research questions: polyvictimization, children or adolescents, and ASD/ADHD diagnosis (see Appendix Table A1). Second, a hand search in reference lists of included papers, conference reports was performed to identify relevant articles that may have been missed in the online search. Searches were also performed in Google and Google Scholar search engines.

### 3.4. Study Selection and Data Collection Process

All identified articles were initially assessed against the inclusion and exclusion criteria. The eligibility of the studies was based on the title and abstract. If the assessments concluded that a study was potentially eligible, the full article was retrieved for further assessment. In the second screening phase, full text papers were assessed independently by the reviewer using a standardized data extraction form designed to describe the characteristics of studies to be included as set out in the recommendations in the Cochrane Handbook Section 5.1.0 https://handbook-5-1.cochrane.org/.

### 3.5. Data Items

Extracted data items included the following characteristics for each study: information about the study sample (age-range, sex, medical diagnosis), country were study was conducted, study design, sample size, survey method, survey informant, types of victimization assessed, prevalence of victimization, frequency (how often victimization occurred), and intensity (severity of the act), if reported.

### 3.6. Quality Assessment

The quality of each study and risk of bias was assessed using the Standard Quality Assessment Criteria For Evaluating Primary Research Papers “QualSyst” [55]. As only quantitative studies are included in this review, the Checklist for Assessing the Quality of Quantitative Studies in QualSyst was used. There are 14 questions in this checklist; for each of which a score of 0 (No), 1 (Partial), or 2 (Yes) can be given. An option of Not Applicable can also be chosen if the question does not apply to the study (for example, a question about randomization is not applicable for a cross-sectional study). A total score is calculated as the sum of all the scores for each question. A summary score is then calculated as the division between the total score and the total possible sum (which is calculated as 28—(number of “not applicable” * 2)). The summary score may range from 0 to 1. In addition to these, an additional item about whether ethics approval had been granted for the conduct of the study is included [34].

## 4. Results

### 4.1. Study Selection

**Figure 1:**
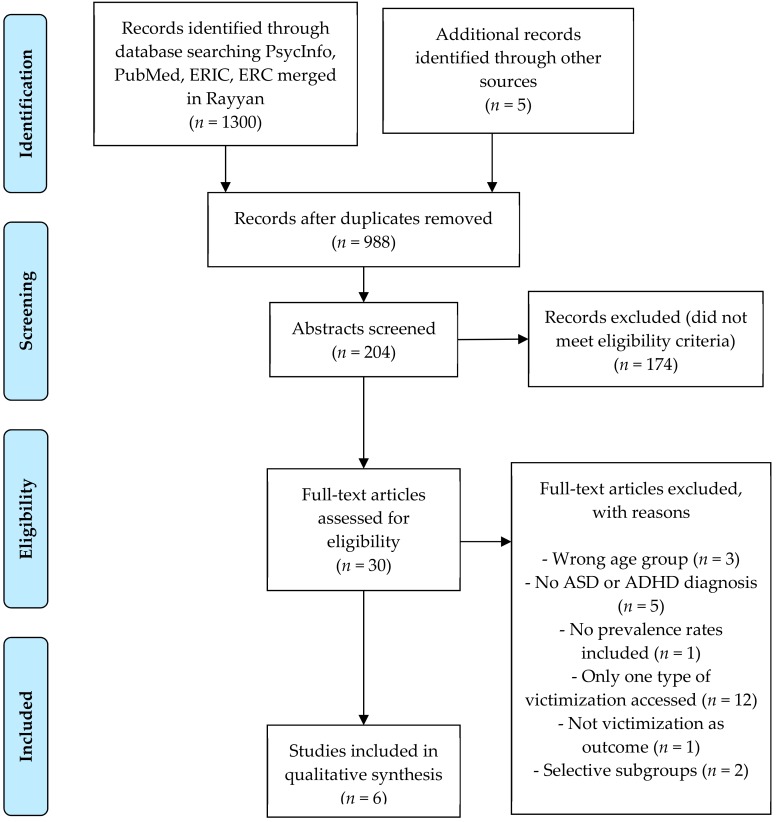
Flow chart diagram showing the process of study selection.

A total of 1300 articles were identified in the data base searches. Five additional records were identified through reference lists. Thirty articles were read in full to determine their inclusion eligibility. Reasons for exclusion included wrong age group (*n* = 3), the target group had no specified ASD or ADHD diagnosis (*n* = 5), no prevalence rates were reported in the results (*n* = 1), only one type of victimization was assessed (*n* = 12), victimization was not reported as the outcome (*n* = 1) and the article used selective subgroups including adolescents with suicidal behavior (*n* = 1) and depression (*n* = 1). Of the 12 articles excluded based on number of victimization types assessed, four examined only peer victimization, six assessed maltreatment and two assessed abuse. A total of six articles met the criteria for inclusion in this review (see Figure 1, flow chart). Together they reported data from 9020 adolescents and adults ranging in sample sizes from 92 to 4114. All studies but one [56] used samples with predominantly men.

### 4.2. Study Characteristics

The characteristics of the included studies are summarized in Table 1.

#### 4.2.1. Study Settings

The five included articles were from USA [7,57], Spain [58], Norway [56], Hong Kong [59], and France [60].

#### 4.2.2. Study Design

Of the studies meeting inclusion criteria, all used a cross-sectional design. One study used children without disabilities as control group [60], two studies used children with other disabilities as control group [7,56], and one study used children with other disabilities and children without disabilities as control groups [59].

#### 4.2.3. Place of Sample Recruitment and Informants

One of the included studies used a population-based design to recruit participants in which they were identified through random selection of nationwide households [7]. The study included 268 respondents with ADHD where the caregiver who “is most familiar with the child’s daily routine and experiences” to children under the age of ten were used as respondents. If the child was over the age of ten, he or she conducted the telephone interview. Chan et al. [59] used a representative sample of 4114 school-aged children with five different types of disabilities. Parents or major caregivers provided proxy reports. Another study recruited 262 respondents through an online networking collaboration for families affected by autism [57]. Due to Institutional Review Board restrictions around the direct questioning of children with disabilities, the study used caregivers as respondents. Three studies used expert centers or welfare institutions to recruit participants. Of these, Aguado-Gracia et al., [58] included 106 outpatients with ADHD in active treatment at the Child and Adolescent Mental Health Center as respondents. The survey in the study was answered by children if they were over the age of 12 and by caregivers if the children were under the age of 12. Greger et al., [56] included 335 adolescents with Asperger’s syndrome or ADHD from child welfare institutions with the adolescents themselves as respondents. Paul et al., [60] recruited their sample consisting of 39 patients with ASD from an ASD expert centre. Due to ethical restrictions, the caregivers were used as respondents.

#### 4.2.4. Types of Diagnosis Examined

Two of the included studies involved respondents with an ADHD diagnosis, two studies involved respondents with an ASD diagnosis, and two studies involved respondents with ADHD or ASD (of which one was explicitly Asperger’s syndrome).

#### 4.2.5. Measures Used for Assessment of Polyvictimization

Among the studies included in this review, the most commonly used assessment tool to examine polyvictimization was the Juvenile Victimization Questionnaire (JVQ). Five studies used JVQ while one study used a modified scale based on the Adverse Childhood Experience (ACE) questionnaire to examine polyvictimization. The ACE questionnaire consist of seven categories of childhood exposures to abuse and household dysfunction [61], whereas the modified version included four categories.

#### 4.2.6. Types of Victimization Examined

Various forms of victimization were examined in the included studies covering many different contexts, i.e., home, school, and community. The forms included differed between studies and were given somewhat different labels depending on what measure was used for assessing polyvictimization. The studies using JVQ included the following forms of victimization: conventional crime (sometimes expressed as property crime), child maltreatment, peer and sibling victimization, sexual assault, witnessing and indirect victimization. The study using the ACE questionnaire included the following forms of victimization: exposure to family violence, exposure to community violence, victim of family violence, victim of community violence and victim of sexual abuse or rape [56].

### 4.3. Risk of Bias Within Studies

In line with previous studies [34], items 5–7 of the QualSyst were not applicable and thus removed from the quality assessment as the included studies were epidemiological and not clinical trials. The total QualSyst score was 22 (see Table 1). Overall, the total sum scores ranged from 17 to 22. Mean score was 20 and summary scores ranged from 0.77 to 1.0 (out of 1.0). Three studies received maximum score (22). All except one study [58], included information about ethics approval. For detailed assessment scores, see Appendix Table A2.

### 4.4. Results of Individual Studies and Synthesis of Results

#### 4.4.1. Prevalence of Specific Forms of Victimization

##### 4.4.1.1 Conventional Crime

All of the six included studies assessed some form of conventional crime. The study by Aguado-Garcia et al. [58] showed an overall prevalence rate of 75.5% for lifetime conventional crime victimization among children with ADHD. The most common form of reported victimization was vandalism (45.3%) and the least common form was kidnapping (1.9%) and bias attack (12.3%). The study by Turner et al. [7] assessed property crime during the past year and found that 38.3% of children with ADHD had experienced this type of victimization while 26.5% of children without ADHD reported the same. Greger et al. [56] assessed victims of community violence and found that 27.5% of children with Asperger’s syndrome reported victimization in the last three months, while 34.6% of children with ADHD reported victimization. Chan et al. [59,62] reported a prevalence rate of 37.1% among children with ADHD and 29.4% among children with ASD in the last year. Among the studies involving children with only an ASD diagnosis, Paul et al. [60] examined lifetime victimization by conventional crime and reported prevalence rates for eight separate items. The most common form of property crime was assault without a weapon, where 56.4% of children with ASD and 49.1% among controls reported being victims, and attempted assault (38.5% ASD, 28.3% controls). The least common form of property crime reported was kidnapping (0%) and robbery (10.3% ASD, 5.7% controls). Pfeffer reported on the same eight items and found that 38.5% of children with ASD reported victimization by assault without a weapon in the past year, while 52.5% reported victimization in their lifetime. A total of 34.4% reported robbery in the last year (49.2% in their lifetime) while 12.9% reported assault with a weapon in the last year (23.5% in their lifetime) and 1.4 reported bias attack in the last year (22.0% in their lifetime).

##### 4.4.1.2. Child Maltreatment

All of the six included studies assessed child maltreatment. Five studies used the JVQ. Two of these studies included only children with ADHD as target group and found an overall prevalence rate of 16.9% for victimization in the last year [7] and 29.2% for lifetime victimization [58]. The prevalence for the different forms of child maltreatment was emotional abuse (18.9%), physical abuse (15.1%), neglect (6.6%), and custodial interference (3.8%) [58]. Chan et al. [59] reported a prevalence rate of 30.0% among children with ADHD and 23.5% among children with ASD in the last year. Two studies using JVQ included only children with ASD as target group and found an overall prevalence rate of 36% (experienced last year) and 50.4% (experienced in a lifetime) [57]. The same study reported prevalence rates of emotional abuse (43.7% for lifetime victimization and 31.6% for experienced last year) and physical abuse (20.6% for lifetime victimization and 11.2% for experienced last year) as the highest for child maltreatment. Paul et al. [60] reported emotional abuse as the most common form of lifetime child maltreatment where 33.3% of children with ASD reported victimization (compared to 20.8% among controls), followed by physical abuse (7.7% ASD, 11.3% controls) and neglect (2.6% ASD, 1.9% controls). Greger et al. [56] assessed victims of family violence and found that 29.9% of children with Asperger’s syndrome reported victimization in the last three months, while 27.1% of children with ADHD reported victimization.

##### 4.4.1.3. Peer and Sibling Victimization

Five of the six studies assessed some form of peer/sibling victimization using JVQ [7,57,58,59,60]. Aguado-Garcia et al. [58] reported a total prevalence rate of 67.9% of lifetime peer/sibling victimization among children with ADHD. Divided by the six different types of victimization, 45.3% of the children reported experienced relational aggression by peers; peer or sibling assault (41.5%); physical intimidation by peer (26.4%); gang or group assault (10.4%); nonsexual genital assault (9.4%); and dating violence (0.9%). Turner et al. [7] report a total prevalence rate of 45.7% of peer assault/bullying in the past year among children with ADHD, compared to 31.5% among children without ADHD. Chan et al. [59,62] reported a prevalence rate of 33.5% among children with ADHD and 21.5% among children with ASD in the last year. Further, the same study reported bullying prevalence rates of 32.1% (ADHD) and 21.0% (ASD) as well as cyberbullying prevalence rates of 32.3% (ADHD) and 22.0% (ASD). In the study by Paul et al. [60], 59% of the parents to children with ASD reported emotional bullying compared to 32.1% among the controls. 66.7% reported peer or sibling assault (60.4% controls); 46.2% reported bullying (11.3% controls); 10.3% report gang or group assault (7.5% controls); 5.1% report nonsexual genital assault (3.8% controls); and 2.6% report dating violence (0% controls). Pfeffer [57] also used parents to children with ASD as informants. In the study, 52.8% of the parents to children with ASD report teasing or emotional bullying in the last year (61.6% lifetime); 32.8% report bullying in the last year (40.4% lifetime); 4.4% report gang/group assault in the last year (9.6% lifetime); and 0.8% report date violence in the last year (2.4% lifetime).

##### 4.4.1.4. Sexual Victimization

All of the studies included assessments of sexual abuse. Among children with ADHD, the prevalence rates ranged from 6.6% from a lifetime perspective [58] and 6.7% from a yearly perspective [7] to 38.2% victimization in the last three months [56]. For respondents with ASD, the study by Greger et al. [56] showed that 27.3% of children with Asperger’s syndrome reported sexual abuse or rape in the last three months, while Pfeffer [57] reported prevalence rates of 7.6% (in the last year) and 14.0% (lifetime). Chan et al. [59] reported sexual abuse among 2.8% of children with ASD in the last year. The most common forms of sexual abuse reported was verbal sexual harassment where the prevalence rates ranged from 2.8% [58] to 8.4% (lifetime victimization) [57], and flashing/sexual exposure were prevalence rates ranged from 0 [58] and 2.6% (5.7% among controls) [60] to 5.2% (lifetime victimization) [57].

##### 4.4.1.5. Witnessing and Indirect Victimization

All of the studies, except for Turner et al. [7], included assessments of witnessing and indirect victimization in the home and in the community. For the two studies examining witnessing of violence among children with ADHD, the overall prevalence rates varied between 41.5% [58] to 47.6% (exposure to family violence) in the last three months [56]. Of these two, only Aguado-Gracia et al. [58] reported specific incidents, where witnessing assault without weapon (22.6%) were the most commonly reported, followed by witnessing assault with weapon (11.3%), burglary (10.4%), and witness to domestic violence (8.5%). One study reported an overall prevalence rate of exposure to witnessing and indirect victimization among children with ASD, were prevalence rates varied between 20.4% (in the last year) to 30.0% (from a lifetime perspective) [57]. Chan et al. [59] reported a prevalence rate of 14.0% among children with ADHD and 5.9% among children with ASD in the last year. Paul et al. [58] and Pfeffer [57] reported the most common form of lifetime exposure to witnessing and indirect victimization among children with ASD as: witness to assault without weapon (28.2% and 15.6%), witness to assault with weapon (12.8% and 7.2%), household theft (15.4% and 16.1%), and witness to domestic violence (2.6% and 8.4%), respectively.

##### 4.4.1.6. Polyvictimization

All six included studies examined at least four different forms of victimization, of which polyvictimization was reported in four [56,57,59,60]. Two studies did not report data on polyvictimization, of which one study aimed to create a better understanding of how specific forms of disability create differential risk for different types of victimization [7] and another to describe the association between frequency and type of victimization with the severity of ADHD symptoms [58]. Greger et al. [56] constructed a victimization scale from 0 to 4 and found a significant relationship between an increasing number of victimization types and the prevalence of Asperger’s syndrome as well as ADHD. Analyzing the number of experienced victimization types as a categorical variable, they found that those who had been exposed to all four adversities had an OR of 3.7 (95% CI: 0.87 to 15.6, *p* = 0.077) for having Asperger’s syndrome, and an OR of 1.79 (95% CI: 0.54 to 6.0, *p* = 0.343) for having ADHD. Paul et al. [60] found that 23.1% (9 out of 39) of ASD students were polyvictims, i.e., reported five or more victimization types within the past year and 11 or more victimization types over a lifetime. However, they found no significant difference compared to the control group of typically developing children. Pfeffer [57] found that children with ASD experiencing at least one form of assault or bullying were at increased risk to experience another type of victimization in the same year. This included maltreatment (RR = 9.77, 95% CI [4.39, 15.58], *p* < 0.000), witnessed or indirect crimes (RR = 3.96, 95% CI [1.63, 7.77], *p* < 0.001), and property crimes (RR = 3.17, 95% CI [2.24, 3.39], *p* < 0.000). Further, of the children who did not experience an incident of assault or bullying within the last year, less than 5% experienced an incident of maltreatment during that period, while 46.5 % of the children who were exposed to assault/bullying also experienced an incident of maltreatment during the last year. Chan et al. [59] examined the association between disabilities and number of child victimization types. Compared to children without disabilities, children with ASD were 1.43 times more likely to report one to three types of victimization (*p* < 0.05) and children with ADHD were 2.62 times more likely to experience four or more types of victimization (*p* < 0.001).

## 5. Discussion

This is the first systematic review of the evidence about polyvictimization among children and adolescents with ASD or ADHD. The included studies represent data from North America (USA), Asia (Hong Kong), and Europe (Spain, Norway, and France). Even though five of the six included studies used the same questionnaire (the Juvenile Victimization Questionnaire), comparison of results was somewhat difficult depending on timeframes, type of respondents (self-reports or parent-reports), and definitions and presentation of the results. The time frame of experiences of victimization varied from the last three months to lifetime. Information about the frequency or intensity of the victimization was either not investigated or not reported. The results regarding polyvictimization among children and adolescents with ASD or ADHD was very scant, indicating that this is a highly under-researched area. Due to the small sample sizes and study design in most studies, it was difficult to draw any confirmatory conclusions regarding differences related to age, gender, and school placement.

### 5.1. Polyvictimization

The overall prevalence of polyvictimization among children with ASD or ADHD could not be estimated in this review due to the small number of studies presenting these types of data. Only two studies reported prevalence rates for polyvictimization. Among children exposed to four types of victimization or more, the reported prevalence rates were 1.8% and 23.1% for children with ASD and 7.3% for children with ADHD [59,60]. There were general tendencies showing that children with ASD or ADHD are exposed to more victimization reported as odds ratios, relative risks, or risk ratios, compared to typically developing children. One study found a strong relationship between bullying and child maltreatment as half of the children reporting bullying also reported child maltreatment [57]. This is confirmed in other research showing a strong association between bullying experiences (both as victim and perpetrator) and child abuse, indicating that bullying appears to be an indicator of severe violence in the home [20]. Considering this strong relationship, it is interesting that only one of the studies included in this review included questions on cyber bullying when measuring sibling/peer victimization [59]. This means that cyberbullying, which is an important arena for victimization, may be unreported when it comes to victimization and polyvictimization among children with ASD and ADHD. A recent review showed that children with neurodevelopmental disorders (including ASD and ADHD) may be involved in cyber bullying to a greater extent compared to typically developing children [18]. However, lack of recognition about new forms of victimization, such as cyberbullying, and their harmful impacts on health and well-being means that parents, teachers, and other people may not be aware of such forms of victimization [34].

### 5.2. Different Forms of Victimization

Among the reported forms of victimization, peer/sibling victimization received the highest prevalence rates, ranging from 21.5–83.3% among children with ASD and 33.5–67.9% among children with ADHD. This is similar to previous research on peer victimization among students with ASD [13] and ADHD [6]. Prevalence rates of peer-victimization among children with ASD and ADHD were only directly compared in one study [59] who found a slightly higher prevalence rate among students (6–18 years) with ADHD in the preceding year. The same study showed an increase risk of most forms of victimization among children in inclusive school settings compared to children in segregated school settings. It is debated whether the inclusion of children with ASD in mainstream schools leads to higher rates of peer-victimization while a segregated school setting could work protectively [5,15,59]. To be able to draw more reliable conclusions, more research on this topic is needed. As suggested previously, frequent peer victimization appears to be an indicator of severe violence at home [20]. Regarding child maltreatment in the current review, in conformity with peer-victimization, children with ASD reported prevalence rates ranging from 16.2–50.4% compared to 16.9–30.0% among children with ADHD. The included studies also showed that children with ASD reported significantly higher prevalence rates for peer and sibling victimization [60] and child maltreatment [59,60] compared to typically developing children. In this review, the lowest prevalence rates for child maltreatment were reported in the studies using parents as respondents [7,60]. Child maltreatment could be a somewhat complicated form of victimization to investigate among children with ASD and ADHD. As parents or caregivers to children with these diagnoses often are the respondents, it could imply a great number of unrecorded cases due to shame or fear of negative consequences of reporting [63]. Further, the prevalence rates for conventional crime, sexual abuse, and witnessing and indirect violence were 34.6–75.5%; 6.6–38.2%; and 14.0–41.5%, respectively, among children with ADHD, and 8.1–64.2%; 2.8–27.3%; and 5.9–30.0%, respectively, for children with ASD. It appears that children with ASD and ADHD are particularly vulnerable to victimization involving the most important and meaningful relationships (peers, siblings, parents or legal guardians). This could be explained by the nature of the ASD and ADHD diagnosis. It has been suggested that children with disabilities may be at greater risk for maltreatment because their extensive needs and/or behavioral difficulties can trigger maladaptive responses in some parents [28]. Children with ASD also tend to have communication deficits, such as responding inappropriately in conversations, misreading nonverbal interactions, or having difficulty building friendships appropriate to their age [3] as well as behavior problems and difficulty with social skills [4]. They may also experience challenges in understanding their own and others’ behavior [64] and have difficulties with self-regulation of their behavior and emotions [65,66]. Further, children with ADHD also exhibit externalizing symptoms, including impulsivity, attention problems, and aggression, which are traits that peers and parents may find aversive and that will lead to difficulties with socialization and caregiving demands [7]. Much of the earlier peer victimization research has identified these characteristics as risk factors for peer victimization involvement and found that children with ASD and ADHD are especially prone to social victimization [48,67]. As Rose and Espelage [68] have argued, it is not the disability itself that is a risk factor but the characteristics associated with the disability.

### 5.3. Type of Respondents

Of the six included studies in this review, only one relied solely on self-reports [56] to measure victimization. Two of the studies reported that ethical restrictions around the direct questioning of children with disabilities prohibited them to perform the assessments with children and youth [57,60]. Some of the studies discussed the use of parental respondents as a study limitation due to recall bias. For example, under-reporting of certain types of victimization, especially victimization in the home and under-reporting due to caregivers’ unawareness of the child’s exposure to certain types of victimization because of ignorance or the unwillingness to share this information. Using self-reports were also discussed as a limitation when selection of youth included only children with high functioning autism and Asperger’s syndrome who were able to fill in the self-report measure used in the study. Due to the nature of the disability, many researchers discuss the reliability of self-reports among children with ASD [15,69]. It is discussed that the symptoms and characteristics of the disability, i.e., difficulties understanding the thoughts, emotions, reactions, and behaviors of others, that makes them the ideal target for bullying may also make it hard for them to perceive and report bullying and victimization in a reliable and valid manner [70]. While many argue that children with ASD are unreliable respondents of victimization, under-reporting using parental and teacher reports have been shown in research on peer victimization [71,72] and child maltreatment [63]. Experiences with peer victimization as reported by ASD youth themselves may differ from those typically measured in questionnaires, indicating that their personal experiences and perceptions of such victimization are not well understood [47]. It may be difficult for children with ASD to differentiate between playful teasing amongst friends and hurtful teasing. Therefore, qualitative methods may be particularly useful in determining how respondents define and understand their own bullying perpetration and victimization experiences [15]. As research has shown that self-report measures associate with internalizing symptoms in children with ASD, using children themselves as informants instead of solely relying on parental reports is important to grasp the full impact of victimization [51].

## 6. Limitations

There are some limitations to be acknowledged. First, the review only included six studies on polyvictimization among children with ASD and ADHD, of which four reported statistics regarding polyvictimization. The sample sizes were in general small, and not all studies used control groups. This indicates that it is difficult to draw any strong conclusions regarding children with ASD or ADHD and their vulnerability to polyvictimization. Second, the inclusion criteria stated that at least four different forms of victimization needed to be investigated in order to be included in the review, meaning that studies examining multiple forms of victimization (i.e., three or two) may have been left out of the study. However, to be able to make valid comparisons between studies, this review was based on the established definition of polyvictimization and in line with previous studies, demanding four different forms of victimization. Third, only two studies included adolescents with ASD or ADHD in the same study, making it hard to draw any conclusions regarding differences between these diagnoses. However, this was not explicitly stated as an aim in the current review. Fourth, three of the included studies used clinical samples meaning that it is difficult to generalize the results to the general population.

## 7. Conclusions

This review presents important information regarding victimization among children with autism spectrum disorder and attention deficit hyperactivity disorder. Accordingly, it highlights the high prevalence of violence and victimization, including polyvictimization, among children and youth with these disabilities. Due to the nature of the diagnoses, these children may be particularly vulnerable to victimization. The review further highlights significant knowledge gaps and important methodological considerations regarding polyvictimization among children with ASD or ADHD, which provide important implications for future research. To be able to enhance the knowledge and improve the life situation for children with ASD and ADHD, comprehensive investigations examining polyvictimization in the contexts of home, school, and community are warranted. Future studies studying victimization in this population should incorporate exposure across different contexts to be able to grasp the full magnitude of victimization. This includes questions on cyber bullying to minimize risk of under reporting, as well as information about the frequency or intensity of victimization to determine the severity and possible negative impacts. This includes information about how often each type of victimization has occurred. It is evident that there are ethical restrictions around the direct questioning of children with disabilities that prohibit researchers to perform the assessments with children and youth [57,60]. However, experiences with peer victimization as reported by ASD youth themselves may differ from those typically measured in questionnaires and parental reports are not always reliable when it comes to child maltreatment and peer victimization (including cyber bullying). Hence, more focus needs to be concentrated on designing valid and reliable measurement methods to explore polyvictimization among children with ASD or ADHD where the children’s own voices are heard and taken into account. Lastly, this review also underscores the need for future research to investigate different health outcomes associated with polyvictimization among population-based samples to provide generalizable results regarding the effects of polyvictimization among children with ASD or ADHD. This would assist in the creation of intervention and prevention programs targeting physical and mental health among this target group.

## Figures and Tables

**Table 1:** Summary of findings of polyvictimization among children and adolescents with Autism Spectrum Disorder or Attention Deficit/Hyperactivity Disorder.

Study ID	Country	Sample & Study Design	Sample Size	Diagnosis^1^	Child/Adolescent Age Range (Years)	Questionnaire and Informants	Time Frame	Type of victimization Examined *	Prevalence Rates and Frequency *	Quality Assessment Score (out of 22)
Aguado-Gracia et al. 2018	Spain	clinical sample	*N* = 106 (77.4% male)	ADHD	6–18 (*M* = 11.2, *SD* = 2.63)	Self-report (age > 12) and parent-reports (age < 12) The Juvenile Victimization Questionnaire	Lifetime	CC, CM, PV, SA WIA	CC (75.5%); CM (29.2%); PV (67.9%); SV (6.6%), WIA (41.5%)	19
Chan et al. 2018	Hong-Kong	Representative sample of school-aged children Cross-sectional	Total *N* = 4114 (ADHD *N* = 433) ASD (*N* = 330) No disabilities (*N* = 3013)	ADHD, ASD	6–18	Parental reports The Juvenile Victimization Questionnaire	Yearly	CC, CM, PV, SA WIA, B, CB	CC (ASD = 29.4% ADHD = 37.1%) CM (ASD = 23.5% ADHD = 30.0%) PV (ASD = 21.5% ADHD = 33.5%) SA (ASD = 2.8% ADHD = 8.1%) WIA (ASD = 5.9% ADHD = 14.0%) B (ASD = 21.0% ADHD = 32.1%) CB (ASD = 22.0% ADHD = 32.3%) Polyvictimization (odds) *Any 1 type* (ASD = 1.52 [1.11–2.08] ADHD = 1.31 [0.95–1.80]) *1–3 types* (ASD = 1.43 [1.02–2.00] ADHD = 1.20 [0.84–1.72]) *4 types or more* (ASD = 1.36 [0.79–2.36] ADHD = 2.62 [1.49–4.32])	22
Greger et al. 2015	Norway	Clinical sample (child welfare institutions)	*N* = 400 of which 23%, (*N* = 75) diagnosed with Asperger’s syndrome (ASD) and 32% (*N* = 106) diagnosed with ADHD	AS, ADHD	13–23	The Adverse Childhood Experiences (ACE) Self-report	Last three months	DM, NV, SA, WFV, WCV	DM (AS = 29.9% ADHD = 27.1%) NV (AS = 27.5% ADHD = 34.6%) SA (AS = 27.3% ADHD = 38.2%) WFV (AS = 30.0% ADHD = 47.6%) WCV (AS = 24.1% ADHD = 0.3%)	22
Paul et al. 2018	France	Clinical sample (ASD expert center)	*N* = 92 of which 39 with ASD (84.6% male) and 53 TD (84.9% male).	ASD	8–18 M = 13.23 (*SD* = 2.96)	The Juvenile Victimization Questionnaire Parental-report	Lifetime	CC, CM, PV, SA WIA (see article for prevalence rates of 34 forms of offenses) VAS, VAH, VE	VAS (75.7%) VAH (16.2%) VE (8.1%) Polyvictimization (23.1% ASD; 17% TD)	18
Pfeffer 2016	USA	Population-based	*N* = 262 (82.3% male)	ASD	5–18 M = 11.07 (*SD* = 3.58)	The Juvenile Victimization Questionnaire Parental-report	Lifetime, yearly	CC, CM, PV, SA WIA	CC (*Yearly:* 49.0% *Lifetime:* 64.2%) CM (*Yearly:* 36.0% *Lifetime:* 50.4%) PV (*Yearly:* 74.3% *Lifetime:* 83.8%) SA (*Yearly:* 7.6% *Lifetime:* 14.0%) WIA (*Yearly:* 20.4% *Lifetime:* 30.0%) Polyvictimization 76% yearly	17
Turner et al. 2011	USA	Population-based	4046 of which 6.7% (*N* =) w ADHD diagnosis	ADHD	2–17 (M = 9.6)	The Juvenile Victimization Questionnaire Parental report (age < 10) Self-report (age < 10)	Yearly	CC, PV, SA, CM	CC (38.3%) CM (16.9%) PV (45.7%) SA (6.7%)	22

^1^ ASD= Autism Spectrum Disorder; ADHD= Attention-Deficit/Hyperactivity Disorder; AS= Asperger’s Syndrome; * B = Bullying; CB = Cyberbullying; CC = Conventional (property) crime; CM = Child maltreatment; DM = Domestic (or family) violence; NV = Neighborhood (or community) violence; PV = Peer (and sibling) victimization; SA = Sexual abuse; WIV = Witnessing and Indirect violence; WFV = Witnessing family violence; WCV = Witnessing community violence; VAS = Victims at school; VAH = Victims at home; VE = Victims elsewhere.

## Appendix A

**Table A1:** Search terms and number of hits in each database.

PubMed (2019-03-04)		
Search	Search terms	Number of hits
#1 Children and Adolescents	“Child”[Mesh] OR “Adolescent”[Mesh] OR “Minors”[Mesh] OR “Puberty”[Mesh] OR “Pediatrics”[Mesh] OR child*[Title/Abstract] OR Pediatric*[Title/Abstract] OR Paediatric*[Title/Abstract] OR adolescen*[Title/Abstract] OR teen*[Title/Abstract] OR young*[Title/Abstract] OR youth*[Title/abstract] OR puberty[Title/Abstract] OR minor*[Title/Abstract] OR juvenile*[Title/Abstract] OR school-age*[Title/Abstract] OR boy*[Title/Abstract] OR girl*[Title/Abstract]	4,028,357
#2 ASD or ADHD	“Neurodevelopmental Disorders”[Mesh]) OR Neurodevelopmental Disorder*[Title/Abstract] OR ADHD[Title/Abstract] OR Attention deficit disorder*[Title/Abstract] OR Autis*[Title/Abstract] OR ASD[Title/Abstract] OR Asperger*[Title/Abstract]	198,221
#3 Polyvictimization/ victimization	“Crime Victims” [Mesh] OR “Exposure to violence” [Mesh] OR victim*[Title/Abstract] OR Exposure to violence*[Title/Abstract]	54,149
#4	#1 AND #2 AND #3	752
**PsycInfo** **(2019-03-01)**		
Search	Search terms	Number of hits
#1 Children and Adolescents	MAINSUBJECT.EXACT(“Elementary School Students”) OR MAINSUBJECT.EXACT(“Primary School Students”) OR MAINSUBJECT.EXACT(“Junior High School Students”) OR MAINSUBJECT.EXACT(“Middle School Students”) OR MAINSUBJECT.EXACT(“High School Students”) OR MAINSUBJECT.EXACT(“Kindergarten Students”) OR MAINSUBJECT.EXACT(“Preschool Students”) OR child* OR adolescen* OR youth OR teen* OR minor* OR young* OR juvenile OR “Elementary School” OR “Primary School” OR “Junior High School” OR “Middle School” OR “High School” OR Kindergarten OR Preschool OR “secondary school”	1,732,513
#2 ASD or ADHD	MAINSUBJECT.EXACT(“Attention Deficit Disorder with Hyperactivity”) OR MAINSUBJECT.EXACT(“Neurodevelopmental Disorders”) OR MAINSUBJECT.EXACT(“Autism Spectrum Disorders”) OR autis* OR adhd OR “Attention Deficit Disorder with Hyperactivity” OR “neurodevelopmental disorder*” OR Asperger OR “Neuropsychiatric disorder*”	134,188
#3 Polyvictimization/ victimization	Polyvictimization OR “poly victimization” OR polyvictimisation OR “poly victimisation” OR MAINSUBJECT.EXACT(“Victimization”) OR MAINSUBJECT.EXACT(“Exposure to Violence”) OR MAINSUBJECT.EXACT(“crime victims”) OR “exposure to violence” OR victimization OR victimisation OR “crime victims” OR victim*	29,595
#4	#1 AND #2 AND #3	346
**ERIC via EBSCO (2019-03-01)**		
Search	Search terms	Number of hits
#1 Children and Adolescents	DE “Children” OR DE “Youth” OR DE “Adolescents” OR child* OR youth OR minor* OR teen* OR young* OR adolescen* OR DE “Elementary School Students” OR DE “Middle School Students” OR DE “Students” OR DE “Junior High School Students” OR DE “High School Students” OR DE “Preschool Children” OR DE “Secondary School Students” OR DE “Kindergarten” OR preschool* OR “Elementary School*” OR “Middle School*” OR “primary school” OR “high school” OR “secondary school” OR kindergarten	667,578
#2 ASD or ADHD	DE “Attention Deficit Hyperactivity Disorder” OR DE “Pervasive Developmental Disorders” OR DE “Autism” OR DE “Asperger Syndrome” OR “Attention Deficit Hyperactivity Disorder*” OR “Pervasive Developmental Disorder*” OR autis* OR adhd OR “neurodevelopmental disorder*” OR “neuropsychiatric disorder*” OR Asperger*	30,406
#3 Polyvictimization/ victimization	DE “Victims of Crime” OR DE “Victims” OR polyvictimization OR “poly victimization” OR polyvictimisation OR “poly victimisation” OR victim*	9467
#4	#1 AND #2 AND #3	66
**ERC via EBESCO** **(2019-03-01)**		
Search	Search terms	Number of hits
#1 Children or Adolescents	DE “Children” OR DE “preschool children” OR DE “Kindergarten children” OR DE “Adolescence” OR DE “Teenagers” OR DE “Young adults” OR DE “Youth” DE “School children” OR DE “MIDDLE school students” OR DE “Students” OR DE “JUNIOR high school students” OR DE “High school students” OR DE “Secondary school students” de “Kindergarten children” OR DE “Elementary schools” OR DE “Primary schools” OR child* OR youth OR minor* OR teen* OR young* OR adolescen* OR preschool OR “Elementary School” OR “Middle School” OR “primary school” OR “high school” OR “secondary school” OR kindergarten	1,128,748
#2 ASD or ADHD	DE “Autism” OR DE “Autism spectrum disorders” OR DE “Autism in adolescence” OR DE “Autism in children” OR DE “Attention-deficit hyperactivity disorder” OR DE “Pervasive developmental disorder not otherwise specified” OR autis* OR adhd OR “attention deficit hyperactivity disorders” OR “neurodevelopmental disorder*” OR “neuropsychiatric disorder*” OR “Pervasive Developmental Disorder*”	55,886
#3 Polyvictimization/ victimization	polyvictimization OR “poly victimization” OR polyvictimisation OR “poly victimisation” OR victim*	22,884
#4	#1 AND #2 AND #3	136

**Table A2:** Detailed quality assessment of included studies.

Study	Ethics Approval	Criteria												
		1 ^a^	2 ^a^	3 ^a^	4 ^a^	8 ^a^	9 ^a^	10 ^a^	11 ^a^	12 ^a^	13 ^a^	14 ^a^	Overall Score ^b^	Average Score ^c^
Aguado-Gracia et al. 2018	?	2	2	1	2	2	1	2	1	2	2	2	19	0.86
Chan et al. 2018	2	2	2	2	2	2	2	2	2	2	2	2	22	1.00
Greger et al. 2015	2	2	2	2	2	2	2	2	2	2	2	2	22	1.00
Paul et al. 2018	2	2	2	2	2	2	1	2	2	1	1	1	18	0.82
Pfeffer 2016	2	2	2	1	2	2	1	2	2	1	1	1	17	0.77
Turner et al. 2011	2	2	2	2	2	2	2	2	2	2	2	2	22	1.00

^a^ 0=No, 1=Partial, 2=Yes & ?=Unclear; ^b^ range 0–22; ^c^ range 0–1; Criterion 1: Sufficiently described objective?; Criterion 2: Study design evident & appropriate?; Criterion 3: Method of subject selection or source of information/ input variables described & appropriate?; Criterion 4: Subject characteristics sufficiently described?; Criterion 8: Outcome & exposure measures well defined & robust to measurement/ misclassification bias? Means of assessment reported?; Criterion 9: Sample size appropriate?; Criterion 10: Analytic methods described/ justified & appropriate?; Criterion 11: Some estimate of variance is reported for the main results?; Criterion 12: Controlled for confounding?; Criterion 13: Results reported in sufficient detail?; Criterion 14: Conclusions supported by the results?
